# Physical activity interventions in European primary schools: a scoping review to create a framework for the design of tailored interventions in European countries

**DOI:** 10.3389/fpubh.2024.1321167

**Published:** 2024-02-08

**Authors:** Alice Porter, Robert Walker, Danielle House, Ruth Salway, Sarah Dawson, Sharea Ijaz, Frank de Vocht, Russell Jago

**Affiliations:** ^1^Population Health Sciences, Bristol Medical School, University of Bristol, Bristol, United Kingdom; ^2^NIHR Bristol Biomedical Research Centre, University Hospitals Bristol and Weston NHS Foundation Trust and University of Bristol, Bristol, United Kingdom; ^3^The National Institute for Health Research, Applied Research Collaboration West (NIHR ARC West), University Hospitals Bristol and Weston NHS Foundation Trust and University of Bristol, Bristol, United Kingdom

**Keywords:** physical activity, children, school-based, primary schools, intervention components

## Abstract

**Introduction:**

Schools provide a unique environment to facilitate physical activity for children. However, many school-based physical activity interventions have not been effective. We propose a new approach, which allows schools to tailor interventions to their specific context. This scoping review aimed to identify intervention components from previous school-based physical activity interventions to form the basis of a tailored approach in a European setting.

**Methods:**

Joanna Briggs Institute guidelines for conducting scoping reviews were followed. European school-based intervention studies aimed at increasing physical activity in children aged 7–11 years published in English since 2015 were included. Databases searched were Ovid Medline, Embase, PsycINFO, Web of Science Social Sciences Citation Index, ERIC and British Education Index. Data was extracted on intervention components, context-related factors (geographical location, school size, child socioeconomic status and ethnicity), feasibility, acceptability and cost-effectiveness. A data-driven framework was developed to summarize the identified intervention components.

**Results:**

79 articles were included, constituting 45 intervention studies. We identified 177 intervention components, which were synthesized into a framework of 60 intervention component types across 11 activity opportunities: six within the school day, three within the extended school day and two within the wider school environment. Interventions most frequently targeted physical education (21%), active and outdoor learning (16%), active breaks (15%), and school-level environmewnt (12%). Of the intervention components, 41% were delivered by school staff, 31% by the research team, and 24% by external organizations. Only 19% of intervention studies reported geographical location and only 10% reported school size. Participant ethnicity and socioeconomic information was reported by 15% and 25%, respectively. Intervention acceptability was reported in 51% of studies, feasibility in 49%, and cost effectiveness in 2%.

**Discussion:**

This review offers a first step in developing a future framework to help schools to develop context-specific, tailored interventions. However, there was a lack of reporting of contextual factors within the included studies, making it difficult to understand the role of context. Future research should seek to measure and report contextual factors, and to better understand the important aspects of context within school-based physical activity.

## Introduction

Physical activity has many positive effects on physical and mental health outcomes during childhood, such as improved cardiorespiratory health and fitness and reduced depressive symptoms, as well as improved cognitive function and academic performance (1, 2). However, a large number of children do not meet the World Health Organization (WHO) recommended average of 60 minutes of moderate-to-vigorous physical activity (MVPA) per day (1, 2), with recent accelerometer data suggesting that only 41% of 10–11 year old children meet the recommendation (3, 4). As we emerge from the COVID-19 pandemic, the way in which children are physically active has changed, with fewer children engaging in unstructured forms of physical activity, such as active play, and an increased dependence on structured activities, such as active school clubs (5–7). As girls and children from lower socio-economic groups have greater challenges in engaging in structured activities, these groups may be at risk of lower than their pre-pandemic levels of physical activity (5–8).

Schools can provide an environment in which physical activity can be equitably promoted (9, 10). Research shows that 13% of variability in weekday MVPA in primary school children on average can be attributable to school-level factors, almost double that of individual factors (11, 12). Therefore, schools can provide an important role in promoting physical activity, especially during the pre-adolescent years (aged 7–11) where physical activity has shown to decline with age (13). However, the majority of school-based physical activity interventions are either ineffective at increasing average MVPA or only yield small improvements (14–16). We have argued that one of the main reasons for this is the lack of focus on school context when designing, implementing, and evaluating school-based physical activity interventions (17). That is, the factors that influence schools as a setting for physical activity interventions (such as cultural, social, economic, environmental), as well as the factors influencing those delivering and receiving the physical activity intervention (such as demographic, socioeconomic) (17). School context can vary significantly from one school to another and potentially influences whether an intervention is successful. Therefore, school-based physical activity interventions that have been deemed “ineffective” as a one-size-fits-all approach in previous research may still offer promising ways to promote children’s physical activity if the intervention components are considered separately and implemented and possibly combined within the appropriate school context. We therefore argue for the rethinking of school-based physical activity intervention studies to focus on context and the need for adaptable interventions that build on what is currently offered by schools (17).

We propose a new flexible school-based physical activity portfolio intervention approach to be delivered in European primary schools (18). This will involve schools selecting intervention components from a framework of components identified from previous studies to create their own school-specific portfolio. The intervention components are defined as the individual elements making up an intervention, while the framework is the resource which collates and presents these components for schools to choose from. The school-specific portfolio is then defined as the combination of intervention components selected by each individual school to meet the local contextual needs of the setting, facilities, priorities, culture, and ethos. The portfolio intervention approach is thus based on the idea that a selection of intervention components allows for a bespoke program for each school.

Recently, tailored interventions and whole school approaches have been developed, which recognize the need for school-specific approaches and alternative ways to effectively promote children’s physical activity. Two recent examples include the Creating Active Schools (CAS) Framework (19–21) and the ACTivity PROmotion via Schools (ACTIPROS) ‘toolbox’ (22) which both provide approaches to work alongside schools to co-design or select physical activity interventions and/or policies that are tailored to school needs. The CAS Framework was developed via stakeholder engagement workshops to highlight opportunities for physical activity within the extended school day and provides a framework for co-designing physical activity policies and interventions with schools, to ensure school ownership and sustainability (19–21). Although, this stakeholder engagement approach has merit in identifying physical activity opportunities, the CAS Framework did not systematically review the published literature, which could also provide useful insight into how best to increase children’s physical activity. The ACTIPROS ‘toolbox’ is an intervention approach whereby schools select from a number of previously identified evidence-based interventions (22). The toolbox was created by systematically identifying previous randomized controlled trials of school-based interventions found to be effective in increasing physical activity and/or cardiorespiratory fitness among 6-11-year-old children (23), which were then mapped onto the WHO Health Promoting Schools Framework (a framework associated with positive health effects when incorporated into intervention development) (24). However, the inclusion of effective interventions only may have limited the number of potentially relevant studies to be included to inform future interventions to increase children’s physical activity, as interventions reported as “ineffective” may have effectiveness in certain contexts. In addition, the inclusion of RCTs only may have also limited inclusion of relevant studies, as there may be important learning from non-randomized intervention studies. It is important to highlight here that while we think that these previous approaches have a lot of merit there is potentially even greater benefit from allowing schools to build an intervention at the component level (i.e., the elements making up the whole intervention), rather than at the higher ‘complete intervention’ level. Yet, there is a lack of available literature related to individual intervention components that is needed to inform our context-specific tailored intervention approach, and it is this gap that we sought to address in this scoping review.

The primary aim of this scoping review was to identify existing physical activity intervention components that could form a portfolio of intervention components for delivery in European primary school settings. We limited our search to studies from 2015 that aimed to increase physical activity among children aged 7–11 years to ensure the most current research was captured. Similarly, as we are focused on components that could be combined to form data-driven portfolios for delivery in a European setting, we limited our search to studies in European schools, as school contexts in other countries, such as school structure, provision, facilities, and physical environment, are likely to differ. Our aims aligned with the rationale for conducting a scoping review, as the interest was in identifying intervention components, rather than assessing efficacy (25). In addition, because our framework will allow schools to build their own tailored school-specific portfolio based on their individual school context, the included intervention studies did not have to report effectiveness or have been reported to be effective at increasing physical activity to form part of our inclusion criteria for the framework. Our secondary aims were to identify if there was evidence of feasibility or acceptability for each component and to identify the resources likely required to implement each component.

## Methods

This review was conducted in accordance with the guidance for conducting scoping reviews as outlined by the Joanna Briggs Institute (JBI) guidelines (26, 27) and the checklist for Preferred Reporting for Systematic Review and Meta-analyses (PRISMA)—extension for Scoping Reviews (28, 29) (Supplementary Table 1). The protocol was published on the Open Science Framework (OSF | PASSPORT) (18) on 31st March 2023.

### Search strategy

A comprehensive search strategy was developed by SD (information specialist), with input from RJ and AP. Search terms were discussed and developed for three concepts: school children, physical activity, and school-based interventions. A study design filter was added so that only experimental studies were identified. Limits were also carefully applied to screen out studies that would definitely not meet our inclusion criteria. The databases Ovid Medline, Embase, PsycINFO, Web of Science Social Science Citation Index, ERIC and British Education Index were searched. Supplementary Table 2 presents the full Medline search strategy. The search strategy was tested by AP and refined by SD. Searches were conducted between April and June 2023.

### Study selection

Table 1 presents the inclusion and exclusion criteria, defined in terms of Population, Concept, Context, and type of publication, in line with scoping review protocol guidance (26). Pilot screening was conducted by AP and discussed with the research team to ensure the eligibility criteria were as comprehensive as possible. Studies of interventions lasting less than 4 weeks were excluded to focus the review on interventions with the potential to make sustainable changes to children’s physical activity levels. Additional exclusion criteria were added after pilot screening, which were not specified in the protocol. These were studies not targeting the provision or knowledge of physical activity (e.g., smartphone bans) and studies focused on use of technology (e.g., apps, virtual reality) because they did not align with our aims of identifying intervention components to inform a portfolio intervention approach to directly target physical activity in children, implementable across a range of schools.

**Table 1:** Eligibility criteria.

Terms	Eligibility criteria	
	Inclusion criteria	Exclusion criteria
Population	Older primary school aged children (7–11 years) attending state funded schools Schools in Europe	Special or private schools Children with chronic conditions (including overweight and obesity) or learning difficulties Schools outside of Europe
Concept	Interventions aiming to increase children’s moderate-to-vigorous physical activity (MVPA)	Interventions aiming to increase MVPA in combination with other health behaviors (e.g., healthy eating) Intervention lasting less than 4 weeks. Studies in which intervention components could not be extracted due to lack of detail.
Context	Interventions targeting physical activity during school term time within the extended school day or across the wider school environment (e.g., within school curriculum, school break times, travel to school, before and after school clubs, homework).	Interventions conducted outside of the extended school day (e.g., in school holidays or the use of school facilities for evening community groups) Interventions that did not directly target the provision or knowledge of physical activity (e.g., smartphone bans) Interventions focused on eHealth or use of technology (e.g., apps, virtual reality, electronic tablets)
Type of publication	Peer-reviewed studies of experimental design (e.g., randomized controlled trials, between-subject, quasi-experimental)	Student theses, conference abstracts, editorials, opinion pieces, reviews, protocols, commentaries Articles not published in English

SD imported titles and abstracts into the reference manager Endnote 20 (30) and removed duplicates. AP uploaded and screened all titles and abstracts in Rayyan (31) and RW independently screened 25% (32). All articles that potentially met the inclusion criteria were included for full-text screening. The full text of articles was then screened against the eligibility criteria by RW, with AP independently screening 25% (32). Where full text articles could not be obtained, authors were contacted. Screening discrepancies were discussed and resolved by AP and RW. The reference lists of all included articles were screened by RW (with AP independently screening 25%) to identify additional studies.

### Data extraction

A standardized Excel spreadsheet was created to extract data. Data extraction was piloted by RW and discussed with the research team, leading to revisions to the original data extraction form. These revisions included extracting data at the study level rather than the intervention component level to align with how study findings were reported (e.g., feasibility and acceptability were reported for the intervention as a whole rather than for the intervention components separately). Due to the lack of data on specific barriers and facilitators to implementation in most studies, we instead extracted data where authors had made suggestions to change or improve the studies. RW independently extracted the data from all studies and AP conducted a 25% data check. Data were extracted by intervention study, drawing from all associated articles (i.e., one intervention may have been associated with a pilot or feasibility trial, full trial, qualitative evaluation and/or process evaluation). Feasibility and acceptability were reported using results from associated qualitative and process evaluations if not reported in the full trial study. We extracted data on intervention characteristics (e.g., country of implementation, intervention description, the number of intervention components included, who delivered the intervention components); study characteristics (e.g., study design, duration of study); study populations (e.g., sample size, gender, ethnicity and socioeconomic status of children); and relevant study findings (e.g., evidence of feasibility, acceptability, cost-effectiveness). The data extraction form is presented in Supplementary Data File 1. Intervention characteristics, study characteristics, study populations and relevant study findings were charted and narratively synthesized in the results section. In line with scoping review guidance, we did not appraise the methodology quality of studies (26).

### Framework development

**Figure 1:**
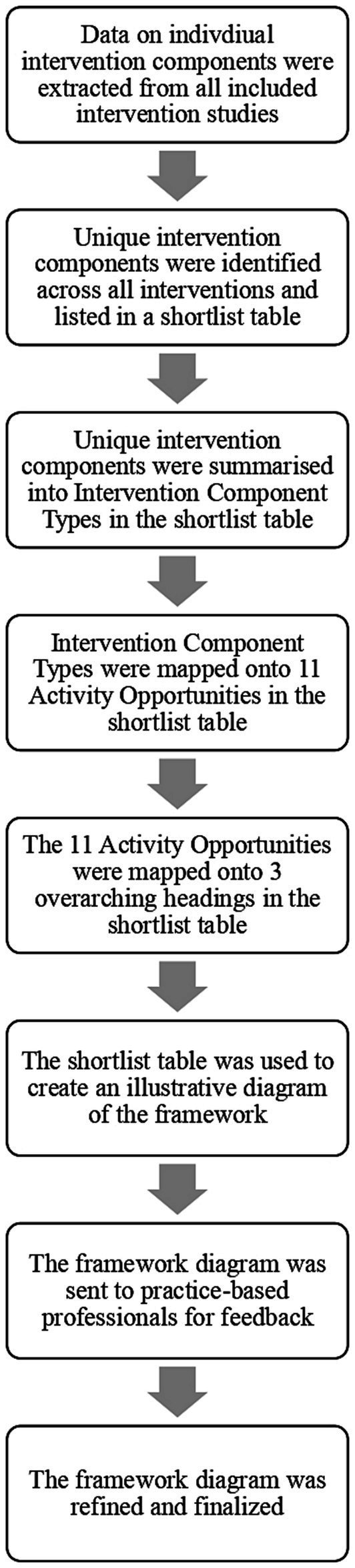
Flow chart of framework development.

**Figure 2:**
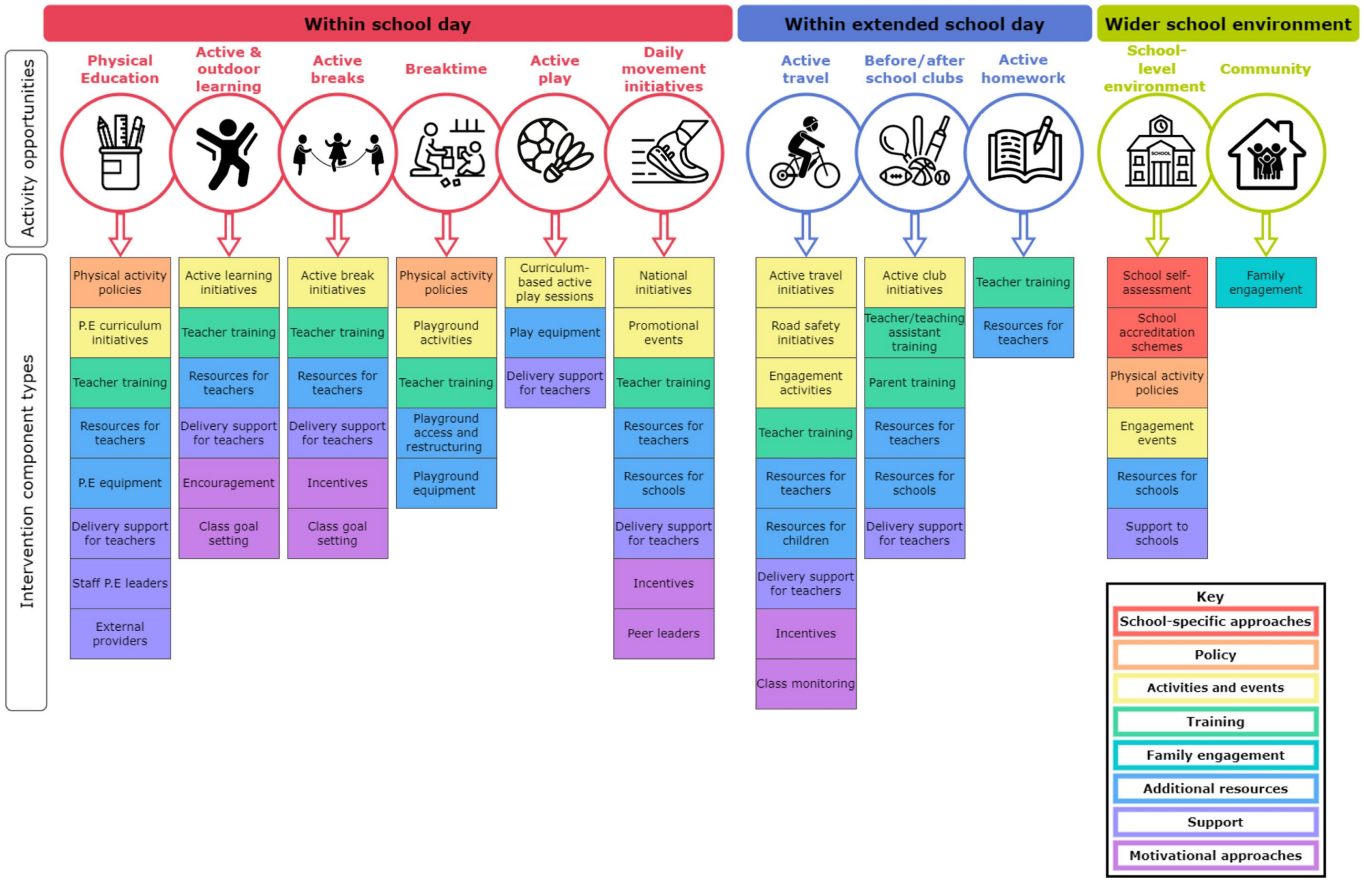
Framework of intervention components.

Data were synthesized into a framework of intervention components. Figure 1 presents a flowchart, which provides an overview of the framework development process. An iterative data-driven approach to framework development was taken via discussions and consensus meetings with the research team, including subject experts and practice-based professionals. Using the data extraction form (Supplementary Data File 1), RW identified the unique intervention components across all interventions. RW then curated a list of intervention components types, which summarized the unique intervention components (e.g., instruction manuals and activity cards were summarized as ‘Resources for teachers’). The intervention component types were then mapped onto an ‘Activity Opportunity’, which was used to highlight which intervention components have been previously used to promote certain physical activity opportunities within schools. The labels and definitions of the activity opportunities were developed using the intervention descriptions in the data extraction form (e.g., the Breaktime activity opportunity was developed from descriptions relating to interventions implemented within school break and lunch times). The activity opportunities were then mapped onto three overarching headings: Within school day; Within extended school day; and Wider school environment, to highlight where in the school system the activity opportunity had been implemented. The intervention component types were color coded to show where the same or similar intervention component types appeared across multiple activity opportunities. Supplementary Data File 2 presents the shortlist of intervention components, highlighting how the unique intervention components were summarized into the higher-level categories described above. Throughout the framework development process, the research team discussed and refined the higher-level categories to ensure clarity. The shortlist was then used to create an illustrative diagram of the framework (Figure 2). The diagram was discussed, drafted and refined by the research team. To increase the external validity of the framework, it was then sent to practice-based professionals, including a multi-academy trust PE strategic lead, a classroom teacher, and a primary education and physical literacy lead at a national children’s physical activity charity for feedback on its appearance and clarity. The framework diagram was further revised after the feedback, which for example included adding additional sub-headings, and editing the language of certain headings.

## Results

**Figure 3:**
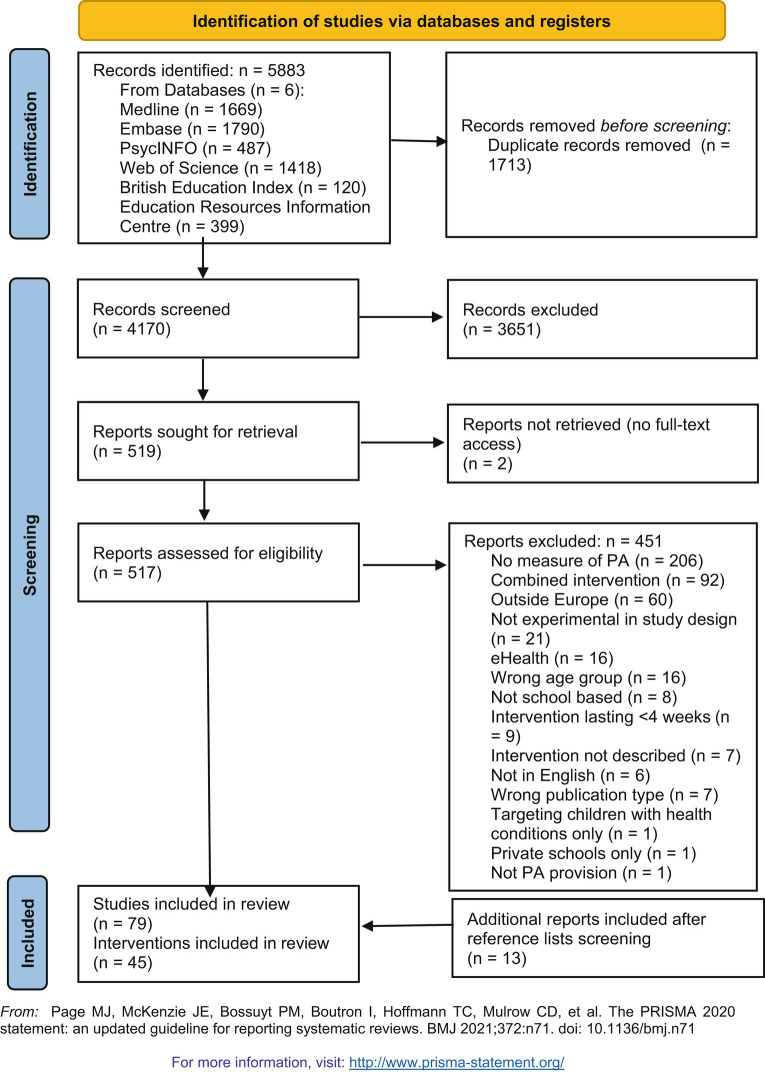
Prisma flow diagram.

We identified 5,883 articles, of which 1,713 were duplicate records. Subsequently, 4,170 were screened at title and abstract level. Of these, 517 articles were screened at full text level, resulting in 79 articles constituting 45 intervention studies (33–112). Figure 3 displays a PRIMSA diagram illustrating detailed information related to screening and inclusion. The detailed data extraction form can be seen in Supplementary Data File 1.

### Intervention characteristics

Table 2 displays the characteristics of the 45 included interventions. Interventions were identified from 11 countries, with interventions implemented in the UK being most common (*n* = 18, 40%). We identified 177 individual intervention components, with between two and five intervention components per intervention being most common (*n* = 32, 71%). Within the 45 interventions, 11 opportunities for physical activity were targeted, with the most frequently occurring being the PE curriculum (*n* = 13, 21%), active and outdoor learning (*n* = 10, 16%), active breaks (*n* = 9, 15%), and school-level environment (*n* = 7, 12%). Members of school staff delivered 72 (41%) of the identified intervention component(s), while the research team and external organizations delivered 54 (31%) and 43 (24%), respectively. The majority of interventions lasted 1–3 months (*n* = 22, 49%), with 16 interventions (36%) lasting longer than 3 months.

**Table 2:** Intervention characteristics.

	Number of studies	%
**Country** ^a^		
UK	18	40.0%
Spain	5	11.1%
Italy	4	8.9%
Ireland	4	8.9%
Finland	3	6.7%
Denmark	3	6.7%
France	2	4.4%
The Netherlands	2	4.4%
Norway	2	4.4%
Germany	1	2.2%
Austria	1	2.2%
**Activity opportunities** ^b^		
PE curriculum	13	21.3%
Active & outdoor learning	10	16.4%
Active breaks	9	14.8%
School-level environment	7	11.5%
Breaktime	6	9.8%
Active travel	5	8.2%
Community	3	4.9%
Daily movement initiatives	3	4.9%
Before/after school clubs	2	3.3%
Active play	2	3.3%
Active homework	2	3.3%
**Number of intervention components** ^a^		
1	4	8.9%
2–3	20	44.4%
4–5	12	26.7%
6–7	4	8.9%
8–9	5	11.1%
**Who delivered the intervention components** ^c^		
School staff	72	40.7%
Research team	54	30.5%
External organizations	43	24.3%
Pupils	1	0.6%
Not specified	18	10.2%
**Intervention duration** ^a^		
1–3 months	22	48.9%
4–6 months	5	11.1%
7–9 months	4	8.9%
10–12 months	5	11.1%
> 1 Year	2	4.4%
Not specified	3	6.7%
No specific duration	4	8.9%

^a^Percentage calculated from the total number of included interventions (*N* = 45).^b^Percentage calculated from the total number of intervention opportunities identified across all studies (note: interventions could include more than one intervention opportunity) (*N* = 61).^c^Percentage calculated from the total number of intervention components (*N* = 177). Note that some components were delivered by multiple people (i.e. research team and school staff).

### Intervention components framework

The intervention components framework is displayed in Figure 2. The 177 individual components identified comprised 100 unique intervention components that were then grouped into 60 broader component types (Supplementary Data File 2). For example, workshops/seminars, CPD opportunities and on the job training for teachers were grouped into “teacher training.” These component types were then mapped to the 11 opportunities to increase physical activity, which are displayed and defined in Table 3. Six activity opportunities were grouped within the school day, those that targeted opportunities during school hours; three within the extended school day, those that targeted opportunities outside of school hours but were linked to the school day; and two within the wider school environment, those that influenced the broader environment or community to promote children’s physical activity. The 60 broader intervention component types were then put into eight categories: (1) activities and events (yellow; 20 unique components); (2) training (green; 13 unique components); (3) additional resources (blue; 29 unique components); (4) support (purple; 14 unique components); (5) motivational approaches (pink; 10 unique components); (6) policy (orange; 5 unique components); (7) school-specific approaches (red; 6 unique components); and (8) family (teal; 3 unique components). As an illustrative example, an intervention component that provided instruction manuals to deliver active learning was categorized as “resources for teachers” and color coded in blue to represent its relationship with other components that provided “additional resources,” which was then displayed under the opportunity “active and outdoor learning” within the larger group “within school day.” The number of components per opportunity for physical activity ranged from three (community) to 15 (Physical Education).

**Table 3:** Activity opportunities and definitions.

	Activity opportunity	Definition
1	PE curriculum	Interventions that made changes to the mandatory school PE curriculum to promote physical activity
2	Active & outdoor learning	Interventions that combined physical activity with non-PE curriculum academic learning objectives to facilitate learning while moving
3	Active breaks	Interventions that used short duration physical activities within the classroom as a break from academic learning
4	Breaktime	Interventions that changed the playground environment to promote physical activity at breaktimes
5	Active play	Interventions that targeted non-breaktime active play (i.e., curriculum time play sessions)
6	Daily movement initiatives	Non-PE curricular programs that regularly encourage children to walk or run over set distances or times, usually taking place outdoors
7	Active travel	Interventions that targeted active modes of travel to and from school (i.e., cycling, walking)
8	Before/after school clubs	Interventions that increased or changed before/after school club provision in order to promote physical activity
9	Active homework	Interventions that used homework with active elements to promote physical activity
10	School-level environment	Interventions that targeted elements of the broader school and its structures to promote physical activity
11	Community	Interventions that drew upon community influences (i.e., the family) to promote physical activity among pupils

### Study design and contextual factors

Information related to study design is displayed in Table 4. We identified three types of experimental design, with most studies being quasi-experimental (*n* = 30, 63%). Baseline sample sizes within the pilot/feasibility studies ranged from 15 to 319 in the experimental groups and 14–165 in control groups. Within the main trials, baseline experimental group sample sizes ranged from 38 to 2,563 and control groups from 22 to 1,343. Mixed methods were employed by 17 (38%) interventions and 6 (13%) had a follow up measure beyond the post-intervention measure.

**Table 4:** Study designs.

	Number of studies	%
**Experimental design** ^a^		
Quasi-experimental	30	62.5%
Randomized controlled trial	15	31.3%
Natural experiment	3	6.3%
**Type of intervention study** ^a^		
Pilot/feasibility study	16	33.3%
Main trial	32	66.7%
Studies which included both	3	6.3%
**Intervention evaluation methods** ^b^		
Quantitative only	28	62.2%
Mixed methods	17	37.8%
**Follow up beyond post-intervention** ^a^		
No follow up	42	87.5%
1–3 months	2	4.2%
4–6 months	3	6.3%
> 6 months	1	2.1%
**Additional evaluations**		
Reported intervention acceptability ^b^	23	51.1%
Reported intervention feasibility ^b^	25	55.6%
Reported cost effectiveness ^b^	1	2.2%

^a^Percentage calculated from the total number of included pilot/feasibility and main trials (*N*= 48). ^b^Percentage calculated from the total number of included interventions (*N* = 45).

Table 5 displays contextual factors reported by intervention pilot/feasibility and main trial evaluations. Few studies reported contextual factors, such as geographical location (*n* = 9, 19%) or school size (*n* = 5, 10%). Seven studies (15%) reported participant ethnicity and 12 reported participant socioeconomic information (25%). Acceptability was reported in 23 (51%) studies, feasibility in 22 (49%), and cost effectiveness in one (2%).

**Table 5:** Study contextual factors.

	Number of studies	%
**School characteristics**		
Reported geographical location	9	18.8%
Reported school size	5	10.4%
**Participant characteristics**		
Reported participant socioeconomic characteristics	12	25.0%
Reported participant ethnicity	7	14.6%

## Discussion

This scoping review has provided a novel synthesis of intervention components that have been reported in European primary school-based physical activity interventions since 2015. We identified 177 individual intervention components that comprised 100 unique components that were then grouped into 60 component types. These components targeted 11 opportunities to increase physical activity, which were categorized into three overarching groups: within the school day; within extended school day; and wider school environment. This information was illustrated in our framework of intervention components (Figure 2). This work forms the basis for creating a portfolio of intervention components that will be used to develop tailored, context-specific school-based physical activity interventions.

The most common opportunities for physical activity targeted by intervention components were PE, active breaks, and active and outdoor learning. This finding aligns with a systematic review and meta-analysis of multi-component school-based physical activity interventions, which identified PE and physical activity during the school day (including active breaks and active learning) as the most common intervention target areas (16). Although a positive trend for the effects of classroom active breaks and active learning has been suggested in the literature, it is challenging to draw conclusions due to low study quality and variability of study designs (113–115). Interventions that target PE have shown to consistently increase in-session physical activity (116–118); however, their impact on whole day physical activity is less clear, with one review finding little positive impact on leisure time physical activity (117). This may be due to compensatory behavior whereby increases in physical activity during one period of the day results in declines in another period (14), emphasizing the need for whole day physical activity measures. Yet, to date, school-based interventions have shown to have a small or no effect on whole day MVPA (14–16). It is clear then that the challenge in increasing MVPA among children requires innovative approaches.

Our results are broadly consistent with a recent scoping review that identified and mapped the characteristics of interventions that sought to increase physical activity or cardiorespiratory fitness among children to the Health Promoting Schools (HPS) framework (23). Aligning with our review, most (58%) interventions centered on health skills and education (i.e., teacher training and materials) and the implementation of active learning, in-class exercises, and improvements to PE, whereas, only 7% of interventions were centered on healthy school policies (23). Although we adopted a data-driven rather than stakeholder-informed approach, the opportunities identified in our review also align with those identified in the Creating Active Schools (CAS) framework (19–21) that include events/visits, break/lunch (recess), PE, curricular lesson, before/afterschool clubs, active travel, and family/community (19–21). Our review provides detailed information related to specific intervention components that can be used to increase physical activity via the opportunities noted in the CAS framework, as well as additional detail to some of the specific opportunities within the CAS opportunities, such as curricular (non-PE) lessons (e.g., active homework, active breaks, daily movement initiatives, and active and outdoor learning). As a result, practitioners may find this information helpful when developing specific approaches.

We have recently proposed a new context-specific approach for school-based physical activity intervention design that emphasizes the varying needs of schools and the subsequent importance of a tailored approach (17). Between-school variability, attributable to unmeasured school factors, has shown to account for nearly double the amount of variation as individual factors (11, 12). Yet, among the studies included in this review, few report descriptive information that can help to understand context, such as geographical location, socioeconomic characteristics, ethnicity, and school size. While this is certainly not an exhaustive list, or even a sufficient level of detail to understand the complexity of school contexts, it reflects what we view as a lack of consideration for contextual factors that are likely to affect intervention effectiveness (17). Collecting relevant data to identify and explore context variation across schools is important to evaluate differential intervention effects, allowing context-specific features to be understood that can be harnessed to promote physical activity. Yet, the aspects of school context that are most important in relation to physical activity is relatively unknown, which makes collecting relevant contextual information challenging. It is therefore important that future research explores school context and its features that influence physical activity.

In our original aim outlined in this scoping review’s protocol (18), we intended to extract detailed information related to the intervention components, including who delivered it, who it was targeted at, resources required, and its duration and frequency. It was our intention that these could subsequently be replicated as part of a portfolio of intervention components that could be developed for individual schools. Yet, it became apparent during extraction that the level of detail needed to be able to replicate components was insufficient. Using teacher training as an example, studies would commonly state the duration and format of the training (i.e., a 1 h workshop), but less often reported the contents of the training sessions being delivered. As a result, researchers and practitioners would be unable to replicate the intervention components reported in these studies. In addition, we were unable to extract resources (e.g., budget, space, number of staff) required to deliver intervention components due to insufficient reporting. This is a well-recognized problem with, for example, a systematic review showing that only 39% of non-pharmaceutical interventions, which included physical activity interventions were adequately described, with missing information related to intervention materials being the most common (47% of studies provided intervention materials) (119). This scoping review adds to this finding and may indicate that inadequate intervention description may be a prevailing issue in physical activity research and steps to improve intervention descriptions might be needed; however, further research to explore this topic in depth on a broader range of studies is needed. Researchers may find the template for intervention description and replication (TIDieR) checklist and guide a useful resource for ensuring interventions are adequately described and reported (120). This would enable researchers to effectively build from the work of others in the field.

Nearly a third (31%) of studies identified in this review were randomized controlled trials (RCTs). These are widely considered the “gold standard” for evaluating interventions (14–16). However, researchers should consider the limitations of RCTs when trying to understand how effectiveness depends on variation between contexts (17). For example, a large number of schools is required to capture the range of contexts in both intervention and control groups to ensure randomization adequately balances contextual differences, which is often not feasible within real-world research that is limited in resources and scope. We have suggested that a cohort-based stepped wedge design could provide an alternative, pragmatic design that allows each school to act as its own control, thus reducing the number of schools needed while maximizing the information available on factors associated with the intervention (17). As such, we suggest that researchers would benefit from considering alternative designs to the RCT in future research.

The cost of implementing school-based interventions varies considerably. For example, in this review we identified an intervention that conducted major playground remodeling (41), which likely comes at relatively greater costs than other interventions, such as changing the way in which PE is taught (46, 47, 75). Cost-effectiveness is therefore an important detail needed to evaluate the effectiveness of physical activity interventions so that informed decisions can be made related to the best use of limited resources. Yet, only one intervention in this review included an evaluation of cost-effectiveness. Including an assessment of cost-effectiveness in future intervention evaluations, where appropriate, is needed to provide additional beneficial information for decision makers and future implementation.

The majority of intervention components identified in this review were delivered by school staff. While a member of school staff may be conveniently placed to deliver an intervention component and more cost-effective to schools than external providers, a lack of time and resources to enable school staff to deliver quality physical activity is a consistent issue identified in the literature (121–123). This issue may have been further exacerbated following the COVID-19 pandemic, where the impact of missed education is evident (124) and schools feel pressured with the need to “catch up” on missed learning while managing the varying post-pandemic needs of each child (125). Therefore, interventions that draw on over-pressured school staff and resources may therefore risk adding further pressure to strained school systems, leading to the intervention not being implemented as intended. This issue was demonstrated pre-pandemic in the process analysis of an intervention included in this review where releasing school staff for training was a key barrier in some schools (54). These systemic pressures within school systems need to be addressed to enable physical activity to be prioritized alongside academic studies within the curriculum. However, researchers and practitioners often have little influence to change these systems and are therefore limited to implementing school-based physical activity interventions within the existing school systems. Systemic pressures likely vary between schools and depend on a number of contextual factors, including school culture, demographics, and community influences. For this reason, context is important, and allowing each school to reflect on their current provision and build intervention components into their specific context, with consideration for their available resources, is vital to promoting physical activity within strained school systems.

The second most common implementer of intervention components was the research team. While these individuals hold expertise in their subject area, this may create delivery agent bias when interventions are scaled up and implemented more widely (126). For example, if the research team are delivering teacher training, when the intervention is scaled up, this training may need to be conducted by a person who does not have the same level of in-depth knowledge or experience as the research team. As a result, the training may be of lower quality and have a less impactful effect on physical activity outcomes. Thus, it would be beneficial to consider the implications of the research team delivering intervention components to ensure that delivery agent bias is minimized when interventions are scaled up. Components that were delivered by the research team also included materials, such as training manuals or guidance. For these materials, researchers may find Patient and Public Involvement (127), or a deeper process of collaboration, a useful means of ensuring that these materials are appropriate for the target population.

The new framework that has been created based on the results of this review does not provide an exhaustive list of intervention components that can be implemented in schools to increase physical activity, but constitute those identified within a specific period of time and population, almost all of which were designed and developed by a research team. This means some potential components and target areas may be missing. For example, through our work with schools, we have seen the implementation of a range of strategies to increase pupil physical activity, such as award ceremonies, t-shirts and other materials to promote school ethos, playground buddy systems, and inspirational school trips to watch sport competitions. Although such interventions are not reported in the academic literature, it is vital that we acknowledge the experientially-informed knowledge of school staff and how these have performed in their specific contexts. Our future research therefore aims to co-design a portfolio of intervention components by synthesizing strategies and interventions developed by both researchers and schools. We envision that this will be completed via workshops and working groups with key stakeholders surrounding children’s school-based physical activity, such as teachers, school senior leadership team members, school governors, and pupils.

Following the co-design workshops, the framework of intervention components will help to facilitate the development of tailored interventions based on the context-specific needs of individual schools. However, there still exists a need to map these components to specific contextual factors. For example, if time and resources are scarce within schools (125), components that require little of each may be appropriate. As discussed above, little is known about primary school contexts and the factors that are most influential to promoting pupil physical activity. Therefore, future work will be needed to combine the framework of intervention components once contextual factors are better understood before it can implemented. It is also important that research is conducted to test the intervention’s efficacy in encouraging children’s physical activity within primary school before it is widely implemented. This work is currently being undertaken as part of the PASSPORT project and will be available once completed and peer-reviewed.

### Strengths and limitations

By mapping the intervention components used in previous European school-based physical activity interventions for children aged 7 to 11 years, this scoping review has provided an initial framework for future intervention development. The resulting framework was data-driven and received input from practice-based professionals to ensure its external validity. The scoping review search strategy was developed by an information specialist and a range of experimental study designs, including natural experiments and quasi-experimental studies were included. In addition, responding to our research highlighting the problematic dismissal of interventions when they do not scale up across contexts or fail to deliver on narrow outcome measures (17), in this scoping review we did not limit our search to interventions found to be effective or successful. However, it is important to highlight the limitations of our scoping review. As highlighted in the discussion, we were unable to extract detailed information about acceptability, feasibility and resource use associated with individual intervention components as we had originally aimed to, due to the lack of reporting across the included studies. We only included studies aiming to increase MVPA and excluded studies exclusively focused on light physical activity, sedentary time or other related health outcomes. Furthermore, we only included studies conducted in European schools and published after 2015 to ensure the intervention components identified were the most relevant for the development of future school-based physical activity interventions in Europe. However, it is possible that studies from other countries, published before 2015 could have provided additional unique components, which could be relevant to European schools. We highlight in the results that 40% of interventions were conducted in the United Kingdom, which may be a reflection of the varying research priorities between countries and there may be interventions published in other languages that were not included in this review. In addition, while we aimed to develop a framework that can be applied across Europe, due to the large number of UK-based interventions, it is warranted to first test the framework in these contexts. Finally, our review was limited to peer-reviewed publications.

## Conclusion

This scoping review has added novel information related to specific intervention components that can be used as a first step in developing a future framework, allowing schools to develop context-specific, tailored interventions to promote children’s physical activity in Europe. This framework addresses a gap in the literature by providing a level of detail at the intervention component level, which is needed to tailor interventions to current school contexts to maximize their capability to promote physical activity. It is important that experientially-informed knowledge is synthesized and included in this framework and co-design workshops with key stakeholders is an important next step in its development. Importantly, we also observed a lack of reporting of contextual factors and cost-effectiveness within the studies included in this review. Future research would benefit from considering these in the design and reporting of school-based physical activity interventions.

## Data availability statement

The original contributions presented in the study are included in the article/Supplementary material, further inquiries can be directed to the corresponding author.

## Author contributions

AP: Conceptualization, Data curation, Methodology, Project administration, Writing – original draft, Writing – review & editing. RW: Conceptualization, Data curation, Methodology, Project administration, Writing – original draft, Writing – review & editing. DH: Conceptualization, Project administration, Writing – review & editing. RS: Conceptualization, Funding acquisition, Methodology, Writing – review & editing. SD: Methodology, Writing – review & editing. SI: Conceptualization, Writing – review & editing. FV: Conceptualization, Writing – review & editing. RJ: Conceptualization, Funding acquisition, Methodology, Writing – review & editing.
