# Supplementary material for: Physical activity interventions in European primary schools: a scoping review to create a framework for the design of tailored interventions in European countries
Source: Front Public Health. 2024 Feb 8;12:1321167. doi: 10.3389/fpubh.2024.1321167 (PMC10883314; doi:10.3389/fpubh.2024.1321167)
Supplement: Supplementary file 2 [file Table_2.DOCX]

**Supplementary Table 2. Medline Search Strategy**

|  | Ovid MEDLINE(R) ALL <1946 to May 15, 2023> |
| --- | --- |
| 1 | Child/ |
| 2 | (child* or boys or girls or kids or juvenil* or minors or paediatric* or pediatric* or young).tw,kf. |
| 3 | 1 or 2 |
| 4 | Schools/ |
| 5 | School Health Services/ |
| 6 | (school* or classroom* or curricul* or teacher or pupils).tw,kf. |
| 7 | or/4-6 |
| 8 | 3 and 7 |
| 9 | schoolchild*.tw,kf. |
| 10 | 8 or 9 |
| 11 | exp Exercise/ |
| 12 | exp Sports/ |
| 13 | exp Physical Fitness/ |
| 14 | Leisure Activities/ or Leisure Time/ |
| 15 | exp Recreation/ |
| 16 | PE lesson?.tw,kf. |
| 17 | (exercise or exercising or games or sport? or sporting or ((leisure or recreation*) adj activ*)).tw,kf. |
| 18 | ((moderate* or graded or vigorous) adj3 activit*).tw,kf. |
| 19 | (walks or walking or daily mile?).tw,kf. |
| 20 | (running or jogging or jumping or hopping or skipping or sprinting or park run? or parkrun* or treadmill? or tread mill? or marathon?).tw,kf. |
| 21 | ((high intensity or HIT or HIIT or circuit) adj2 training).tw,kf. |
| 22 | (bike? or biking or bicycl* or ((recreational or distance) adj cycling)).tw,kf. |
| 23 | (scooter? or skateboard? or skat* board? or skat* park? or skating).tw,kf. |
| 24 | swimming.tw,kf. |
| 25 | (team game? or team sport? or football* or rugby or cricket or hockey or rounders or baseball or basketball or netball or volleyball or handball or tennis or squash or badminton).tw,kf. |
| 26 | (physical adj (activit* or conditioning or training)).tw,kf. |
| 27 | (aerobics or keep* fit or fitness training).tw,kf. |
| 28 | (ballet or dance or dancing or salsa or zumba).tw,kf. |
| 29 | (school gym or gym club or gymnasium? or gymnastics or ((fitness or leisure or wellness) adj (cent* or facility or facilities))).tw,kf. |
| 30 | exercise movement techniques/ or breathing exercises/ or qigong/ or dance therapy/ or tai ji/ or yoga/ or kinesiology/ |
| 31 | (qigong or qi gong or ch'i kung or Tai Chi or Taiji or Tai Chi Chuan or Taichi Quan or Taijiquan or Shadowboxing or Shadow Boxing or Tai Chi Chih or T'ai Chi Chuan or yoga or yogic or pilates or kinesiology).tw,kf. |
| 32 | (martial art? or aikido or judo or Jujutsu or Jujitsu or Ju-Jitsu or Karate or Kickboxing or Taekwondo or Tae Kwon Do or Taekwon-Do).tw,kf. |
| 33 | or/11-32 |
| 34 | 10 and 33 |
| 35 | "physical education and training"/ |
| 36 | teacher training/ or inservice training/ |
| 37 | ((staff or teacher? on inservice or in-service) adj training).tw,kf. |
| 38 | train-the-trainer.tw,kf. |
| 39 | health knowledge, attitudes, practice/ |
| 40 | ((health or wellbeing or well being) adj (attitude? or knowledge)).tw,kf. |
| 41 | health education/ |
| 42 | education.tw,kf. |
| 43 | (educational adj (activiti* or exercis* or game? or project? or program*)).tw,kf. |
| 44 | *motivation/ |
| 45 | health literacy/ |
| 46 | literacy.tw,kf. |
| 47 | exp health promotion/ |
| 48 | (health* adj1 promot*).tw,kf. |
| 49 | health plan implementation/ |
| 50 | lesson plan*.tw,kf. |
| 51 | environment design/ |
| 52 | infrastructure.tw,kf. |
| 53 | ((school* or classroom* or environment* or playground* or play* ground*) adj design*).tw,kf. |
| 54 | ((playground* or play* ground*) adj (based or characteristics or games or markings or project? or program*)).tw,kf. |
| 55 | (school* adj3 (intervention? or plan or plans or policy or policies or practice?)).tw,kf. |
| 56 | (school* adj3 based adj3 (initiative? or intervention? or project? or program* or strateg*)).tw,kf. |
| 57 | ((teacher* or assistant*) adj (deliver* or facilitat* or led)).tw,kf. |
| 58 | (buddy or buddies or (peer? adj3 (based or deliver* or facilitat* or led))).tw,kf. |
| 59 | (confidence adj1 building).tw,kf. |
| 60 | ((activ* or exercis*) adj2 (policy or policies or strateg*)).tw,kf. |
| 61 | (intervention or campaign).tw,kf,hw. |
| 62 | "active for life".tw,kf. |
| 63 | (life* adj2 skills).tw,kf. |
| 64 | (activ* adj1 school*).tw,kf. |
| 65 | (play time? or (activ* adj (break* or recess or play*)) or ((activit* or play* or recreational) adj equipment?)).tw,kf. |
| 66 | ((before or after) adj3 (class or classes or school) adj3 (activ* or club? or program*)).tw,kf. |
| 67 | ((beforeschool or afterschool) adj3 (activ* or club? or program*)).tw,kf. |
| 68 | ((breakfast or activit*) adj club?).tw,kf. |
| 69 | fitness trackers/ |
| 70 | (pedomet* or fitbit? or fit bit? or ((fitness or activit*) adj track*) or (steps adj2 (count* or track* or monitor*))).tw,kf. |
| 71 | transportation/ |
| 72 | travel plan*.tw,kf. |
| 73 | (activ* adj3 (transport* or travel* or commuting or commute*)).tw,kf. |
| 74 | (school* adj5 (transport* or dropoff? or drop-off? or pickup? or pick-up?)).tw,kf. |
| 75 | ((activity adj2 (increas* or measur* or monitor*)) not (drug activity or active drug?)).tw,kf. |
| 76 | (multi* component or multicomponent? or multi* facet* or multifacet* or multi* factor* or multifactor* or multi* level* or multilevel* or multi* modal* or multimodal* or multi* dimension* or multidimension* or multi* disciplin* or multidisciplin*).tw,kf. |
| 77 | or/35-76 |
| 78 | 34 and 77 |
| 79 | Randomized Controlled Trial.pt. |
| 80 | Randomization/ |
| 81 | Random Allocation/ |
| 82 | Controlled Clinical Trial.pt. |
| 83 | (randomi#ed or randomi#ation or randomi#ing).tw,kf. |
| 84 | (RCT or cRCT or "at random" or (random* adj3 (administ* or allocat* or assign* or class* or cluster or crossover or cross-over or control* or determine* or divide* or division or distribut* or expose* or fashion or group* or number* or place* or pragmatic or quasi or recruit* or split or substitut* or treat*))).tw,kf. |
| 85 | trial.ti. |
| 86 | intervention.ti. or (intervention and control*).ab,kf. |
| 87 | ((intervention* or control* or compar*) adj4 group*).ab. |
| 88 | ((control* or compar*) and ((usual adj2 (activit* or practice?)) or "as usual")).ab. |
| 89 | (control* and (trial or study)).tw,kf. |
| 90 | ((single or multi*) adj cent* adj (study or trial)).tw,kf. |
| 91 | Feasibility Studies/ |
| 92 | "Pilot Projects"/ |
| 93 | ((feasibilit* or pilot) adj (study or project? or program*)).tw,kf. |
| 94 | quasi*.tw,kf. |
| 95 | factorial.tw,kf. |
| 96 | attention-control.tw,kf. |
| 97 | controlled before-after studies/ |
| 98 | "controlled before and after".tw,kf. |
| 99 | interrupted time series analysis/ |
| 100 | (time series or time point? or repeat* measur*).tw,kf. |
| 101 | (pre-intervention? or preintervention? or "pre intervention?" or post-intervention? or postintervention? or "post intervention?").tw,kf. |
| 102 | ((before adj5 after) or pre-post or (pre adj5 post) or ((pretest or "pre test") and (posttest or "post test"))).tw,kf. |
| 103 | (control* and (before adj10 (after or during))).tw,kf. |
| 104 | (pre-study or pre-program* or pre-project? or pre-campaign? or pre-initiative? or pre-mandat* or pre-strateg* or post-study or post-program* or post-project? or post-campaign? or post-initiative? or post-mandat* or post-strateg* or ((before or after) adj3 (pilot or program* or project? or campaign? or initiative? or mandat* or strateg*))).tw,kf. |
| 105 | or/79-104 |
| 106 | 78 and 105 |
| 107 | limit 106 to yr="2015 -Current" |
| 108 | (infant* or adolesc* or teen* or young adult*).ti,bt,hw. not child*.mp. |
| 109 | ((pre-school* or preschool*) not (primary school* or junior* or elementary school*)).ti,bt. |
| 110 | ((infant school* or middle school* or high school* or secondary school*) not (primary school* or junior* or elementary school*)).ti,bt. |
| 111 | (asperger* or autism or autistic or ((attention-deficit or hyperactivity) adj2 disorder?) or ADHD or arthriti* or asthma* or cerebral palsy or cystic fibrosis or diabetes or diabetic or epilepsy or epileptic? or ((heart or lung) adj3 (disease? or disorder?)) or cancer or chemo* or (child* adj3 (illness* or disease? or disorder?)) or lympho* or leuk?emi* or multiple sclerosis).ti,bt. |
| 112 | ((physical* or mental* or developmental* or intellectual* or learning or neuro*) adj3 (disease? or disorder? or disabled or disabilit*)).ti,bt. |
| 113 | (Special Education* adj3 (need? or service?)).ti,bt. |
| 114 | or/108-113 |
| 115 | 107 not 114 |
| 116 | exp africa/ or exp asia/ or exp americas/ or exp oceania/ |
| 117 | exp Medicare/ or exp Medicaid/ |
| 118 | (united states or usa or "u.s.a." or "u.s." or veteran* or alabama or montgomery or alaska or juneau or anchorage or arizona or phoenix or arkansas or little rock or california or sacramento or los angeles or colorado or denver or connecticut or hartford or bridgeport or delaware or dover or wilmington or florida or tallahassee or jacksonville or miami or atlanta or hawai?i or honolulu or idaho or boise or illinois or springfield or chicago or indiana or indianapolis or iowa or des moines or kansas or topeka or wichita or kentucky or frankfort or louisville or louisiana or baton rouge or new orleans or maine or augusta or portland or maryland or annapolis or baltimore or massachusetts or boston or michigan or lansing or detroit or minnesota or st paul or minneapolis or mississippi or jackson or missouri or jefferson city or montana or billings or nebraska or omaha or nevada or carson city or las vegas or new hampshire or concord or new jersey or trenton or newark or new mexico or santa fe or albuquerque or new york or albany or north carolina or raleigh or north dakota or bismarck or fargo or ohio or columbus or oklahoma or oregon or salem or pennsylvania or harrisburg or philadelphia or rhode island or providence or south carolina or columbia or charleston or south dakota or sioux falls or tennessee or nashville or texas or austin or houston or utah or salt lake city or vermont or montpelier or burlington or virginia or richmond or washington or olympia or seattle or wisconsin or madison or milwaukee or wyoming or cheyenne).tw,in. |
| 119 | (african american* or hispanic american* or appalachia* or great lake* or medicare or medicaid or mid?atlantic or new england or pacific state*).tw. |
| 120 | or/116-119 |
| 121 | exp Europe/ or European Union/ |
| 122 | (Europe* or Albania* or Andorra* or Austria* or Belarus* or Belgium* or Belgen* or Bosnia* or Herzegovina* or Bulgaria* or Croatia* or Czech* or Cyprus or Cypria* or Denmark* or Danish or Estonia* or Faroe Island* or Finland* or Finnish or France* or French or German* or Greece* or Greek* or Hungary* or Hungarian* or Iceland* or Ireland* or Irish or Italy* or Italian* or Kosovo* or Latvia* or Liechtenstein* or Lithuania* or Luxembourg* or Malta* or Maltese* or Moldova* or Monaco* or Montenegro* or Netherlands* or Dutch or Macedonia* or Norway* or Norwegian* or Poland* or Polish or Portugal* or Portuguese or Romania* or San Marino* or Serbia* or Slovakia* or Slovenia* or Spain* or Spanish or Sweden* or Swedish or Switzerland* or Swiss or Turkey or Turkish or Ukrain* or United Kingdom* or UK or (England not New England) or English or Scotland or Scottish or (Wales not New South Wales) or Welsh).tw,in. |
| 123 | 121 or 122 |
| 124 | 120 not 123 |
| 125 | 115 not 124 |
